# McMahon Score as a predictor of mortality in lightning-associated rhabdomyolysis

**DOI:** 10.1186/s12882-025-04497-2

**Published:** 2025-10-07

**Authors:** Abdullah Şen, Mahmut Yaman, Şilan Göger Ülgüt, Sema Belek, Berçem Tugay Günel, Hasan Mansur Durgun, Mehmet Üstündağ, Ercan Gündüz, Murat Orak, Cahfer Güloğlu

**Affiliations:** 1 Department of Emergency Medicine, Dicle University Faculty of Medicine, Diyarbakır, 21280 Turkey; 2 Department of Emergency Medicine, University of Health Sciences, Diyarbakır Gazi Yasargil Training and Research Hospital, Diyarbakır, 21070 Turkey; 3 Department of Emergency Medicine, Internal Medicine, Dicle University Faculty of Medicine, Diyarbakır, 21280 Turkey

**Keywords:** Acute kidney injury, Lightning strike, McMahon Score, Mortality, Rhabdomyolysis

## Abstract

**Background:**

Lightning strikes are rare but potentially fatal environmental events that can lead to rhabdomyolysis through extensive muscle injury. This condition may result in serious complications such as acute kidney injury (AKI) and death. While the McMahon Score is a recognized prognostic tool in trauma-induced rhabdomyolysis, its effectiveness in lightning-related cases remains uncertain. This study aims to evaluate the prognostic utility of the McMahon Score in predicting mortality and the development of AKI in patients presenting with lightning strike-induced rhabdomyolysis.

**Methods:**

This retrospective cohort study included 66 patients admitted to a tertiary emergency department (ED) after lightning strikes between January 2013 and January 2023. Demographic, clinical, and laboratory data were reviewed. Patients were categorized by McMahon Score (≤ 6 and > 6). Outcomes such as mortality and AKI were compared. Predictive performance was assessed using ROC curve analysis.

**Results:**

The median age was 21 years; 72.7% were male. The overall mortality rate was 21.2%, and AKI incidence was 7.0%. Patients with McMahon Scores > 6 had significantly higher mortality and AKI rates (*p* < 0.05). The score showed strong predictive power for mortality (AUC: 0.951). A cutoff value of 6 yielded 71.4% sensitivity and 100% specificity.

**Conclusions:**

The McMahon Score is a reliable tool for predicting mortality and AKI in lightning-induced rhabdomyolysis. Its simplicity and high accuracy make it valuable for rapid risk assessment in emergency settings.

## Introduction

Lightning strikes are among the rare environmental injuries that can lead to severe complications. Rhabdomyolysis associated with lightning strikes results from the destruction of muscle tissue due to electrical and thermal damage to cell membranes. This process leads to the systemic release of intracellular components such as myoglobin, potassium, and creatine kinase, causing various organ dysfunctions, particularly acute kidney injury (AKI) [1, 2]. The systemic effects of rhabdomyolysis pose significant challenges in the clinical management of lightning strike cases [3]. In this context, prognostic tools are of great importance for determining rapid and effective treatment approaches in such patients. The McMahon Score, a tool used to predict the severity and potential outcomes of rhabdomyolysis, has demonstrated efficacy across various patient populations as reported in the literature [4, 5]. This study aims to evaluate the prognostic value of the McMahon Score in cases of rhabdomyolysis associated with lightning strikes. Rhabdomyolysis is recognized as one of the most common and serious outcomes of complications due to lightning strikes. Although the incidence of rhabdomyolysis secondary to lightning strikes is rarely reported in the literature, it has been shown to be a critical factor influencing the prognosis of such events [6, 7]. The McMahon Score considers parameters such as age, creatine kinase levels, phosphate, and bicarbonate to determine the severity of rhabdomyolysis. Its use plays a crucial role in the early prediction of rhabdomyolysis-associated complications and the optimization of treatment strategies [4, 8]. However, studies on the effectiveness of the McMahon Score in rhabdomyolysis due to lightning strikes are limited. Existing studies have predominantly focused on cases of traumatic rhabdomyolysis, and the impact of environmental factors, such as lightning strikes, on outcomes has not been adequately investigated [9, 10].

In this context, the present study investigates the prognostic utility of the McMahon Score in patients with lightning-induced rhabdomyolysis by examining its relationship with adverse clinical outcomes, particularly mortality and acute kidney injury. By analyzing clinical and laboratory parameters alongside McMahon Score stratification, this study aims to contribute evidence for its applicability in lightning-related muscle injury cases. This study aims not only to evaluate the prognostic value of the McMahon Score in lightning-induced rhabdomyolysis, but also to provide evidence that may support early triage strategies, improve outcome prediction, and guide emergency department management policies in environmental trauma cases.

## Methods

### Study population and design

**Fig. 1:**
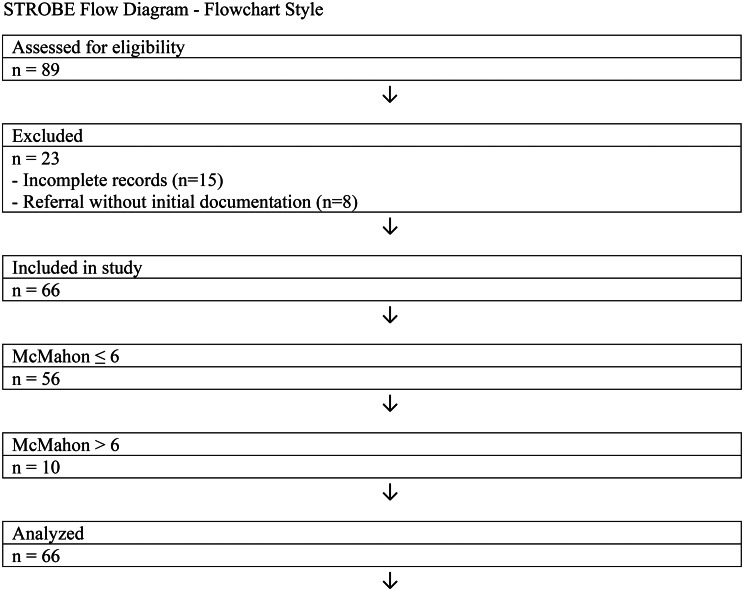
STROBE-style patient selection flow diagram for the lightning-induced rhabdomyolysis cohort (flowchart style)

In the medical records of patients who presented to the emergency department due to lightning strikes, we aimed to evaluate the clinical characteristics, laboratory parameters, and outcomes of patients during the specified period. The study population consisted of patients with a confirmed history of lightning strikes as the primary cause of their ED visit. The inclusion criteria required documented evidence of lightning strike exposure, presentation to the emergency department within 24 h of the incident, and the availability of complete clinical and laboratory records. Patients were excluded if their medical records were incomplete, if they were transferred from other healthcare facilities without sufficient documentation of their initial presentation, or if the lightning strike was not the primary cause of their injury or illness. A total of 66 patients meeting the inclusion criteria were enrolled in the study. A total of 89 patients were screened during the study period. Of these, 23 were excluded (15 due to incomplete data and 8 due to referral without initial records), leaving 66 patients who met the inclusion criteria and were enrolled in the study. A STROBE-compliant patient flow diagram is presented in Fig. 1 to illustrate the selection process. Demographic data, clinical presentation, Glasgow Coma Scale (GCS) scores, laboratory values, and outcomes, including mortality and acute kidney injury (AKI), were retrieved from electronic medical records. Lightning strike injuries were defined according to the International Classification of Diseases (ICD-10) codes associated with environmental injuries. All data were obtained from electronic hospital records and verified against discharge summaries.

### Data collection and variables

All data were anonymized prior to analysis to maintain patient confidentiality. The data collection process was conducted by two independent researchers to ensure accuracy and consistency. Extracted variables included demographic characteristics (age, sex), clinical parameters (GCS score, McMahon Score, presence of AKI), and laboratory findings (hemoglobin, creatinine, creatine kinase, bicarbonate, calcium, and phosphate levels). In this study, day peak creatine kinase was defined as the day during hospitalization on which the highest CK value was measured, regardless of whether it occurred within the first 24 h or later. AKI was defined according to the Kidney Disease: Improving Global Outcomes (KDIGO) criteria as an increase in serum creatinine ≥ 0.3 mg/dL within 48 h or ≥ 1.5 times baseline within 7 days. The term ‘risk of AKI’ in our results refers to the predicted probability of developing AKI as calculated by the McMahon score. Mortality data were derived from hospital records and cross-checked against discharge summaries. All laboratory parameters, including serum phosphate and calcium, were routinely collected for all patients according to the emergency department protocol. Missing data were minimal (< 5%), and patients with missing values were excluded from the corresponding subgroup analyses. The primary outcome was in-hospital mortality, and secondary outcomes included the incidence of AKI and the prognostic value of the McMahon score. The McMahon score was calculated for all patients using standardized criteria, and a cut off value of 6 was utilized for subgroup analysis. Patients were stratified into two groups: those with McMahon scores ≤ 6 and > 6 (Table 1).

**Table 1:** McMahon Score

Variable	Value Range / Condition	Points
Age (years)	50–70 / 71–80 / >80	1.5 / 2.5 / 3
Women	Female	1
Admission creatinine (mmol/L)	124–194 / >194	1.5 / 3
Admission calcium (mmol/L)	< 1.875	2
Admission creatine kinase (U/L)	> 40,000	2
Aetiology	Other than seizures, syncope, exercise, statins, or myositis	3
Initial phosphate (mmol/L)	1.3–1.74 / >1.74	1.5 / 3
Initial bicarbonate (mmol/L)	< 19	2

### Statistical analysis

Continuous and ordinal variables were summarized as medians with interquartile ranges (IQR), while categorical variables were expressed as frequencies and percentages. Comparative analyses between groups (e.g., mortality vs. non-mortality, McMahon ≤ 6 vs. >6) were performed using the chi-square (χ²) test for categorical variables, the Mann–Whitney U test for non-normally distributed continuous variables, and the independent t-test for normally distributed continuous variables. The prognostic accuracy of the McMahon score was evaluated using receiver operating characteristic (ROC) curve analysis, with the area under the curve (AUC) reported. Sensitivity, specificity, positive predictive value (PPV), and negative predictive value (NPV) were calculated for the selected cutoff point of 6. SPSS 28.0 was used for statistical analyses. A two-tailed p-value < 0.05 was considered statistically significant. All p-values and the corresponding test statistics are reported in the tables.

## Results

A total of 66 patients were included in the study. The age of the patients ranged from 16 to 55 years, with a median age of 21 years and a mean age of 24.1 ± 13.3 years. The patient selection process is summarized in a STROBE-compliant flow diagram (Fig. 1).

Of the participants, 72.7% were male. The median Glasgow Coma Scale (GCS) score was determined to be 15. The median value of the McMahon Score was calculated as 4.5, and 84.8% of the patients were classified as having a McMahon Score ≤ 6. Laboratory results showed mean hemoglobin and albumin levels of 14.3 ± 2.0 g/dL and 3.9 ± 0.6 g/dL, respectively. A wide distribution was observed in creatine kinase levels, with a mean peak value of 10240.7 ± 17600.5 U/L. The most frequent day for observing peak creatine kinase was the first day (median: 1.0 day). The mean risk of acute kidney injury (AKI) was determined to be 7.0 ± 8.8%. The mortality rate was calculated as 21.2%. The primary causes of death were cardiac arrest (*n* = 9) and multi-organ failure (*n* = 5) (Table 2).

**Table 2:** Demographic, clinical, and laboratory findings in lightning-induced rhabdomyolysis cases

		Min-Max	Median [IQR]	Median	*p*	
Age		0,0–55,0	14,5–33,0	21,0	***0***,***926***	^t^
Gender	Male (42/6)				***0***,***111***	^X²^
	Female (10/8)					
GCS Score		3,0–15,0	15,0–15,0	15,0	***0***,***000***	^m^
McMahon		0,0–11,5	3,0–5,0	4,5	***0***,***000***	^m^
McMahon Score	< 6			56	***0***,***000***	^X²^
	≥ 6			10		
Hemoglobin		10,2–17,9	13,1–16,0	14,6	***0***,***851***	^t^
Albumin		2,30 − 5,20	3,50 − 4,29	4,07	***0***,***036***	^t^
Lymphocyte		0,88 − 7,27	1,65 − 4,62	2,42	***0***,***466***	^m^
Platelet		118,0-428,3	224,1-339,4	267,0	***0***,***567***	^t^
Phoshpate		2,0–5,0	3,1–4,2	3,7	***0***,***268***	^m^
Calcium		1,1–10,6	8,8–9,9	9,2	***0***,***017***	^m^
Bicarbonate		4,3–28,0	18,8–24,4	22,4	***0***,***002***	^m^
Creatinine		0,33 − 3,22	0,67 − 1,04	0,83	***0***,***004***	^m^
Creatine Kinase		86-84500	1680–6520	2550	***0***,***002***	^m^
Peak Creatine Kinase		86-84500	1560–10,230	3330	***0***,***004***	^m^
Day Peak Creatine Kinase		1,0–7,0	1,0–2,0	1,0	***0***,***007***	^m^
% Risk of Acute Kidney Injury		1,0–50,9	3,2–7,0	5,8	***0***,***000***	^m^
Mortality	(-)			52(78.8)		
	(+)			14(21.2)		

^t^ Independent t test / ^m^ Mann-whitney u test / ^X²^ Chi-square test (Fischer test)

**Fig. 2:**
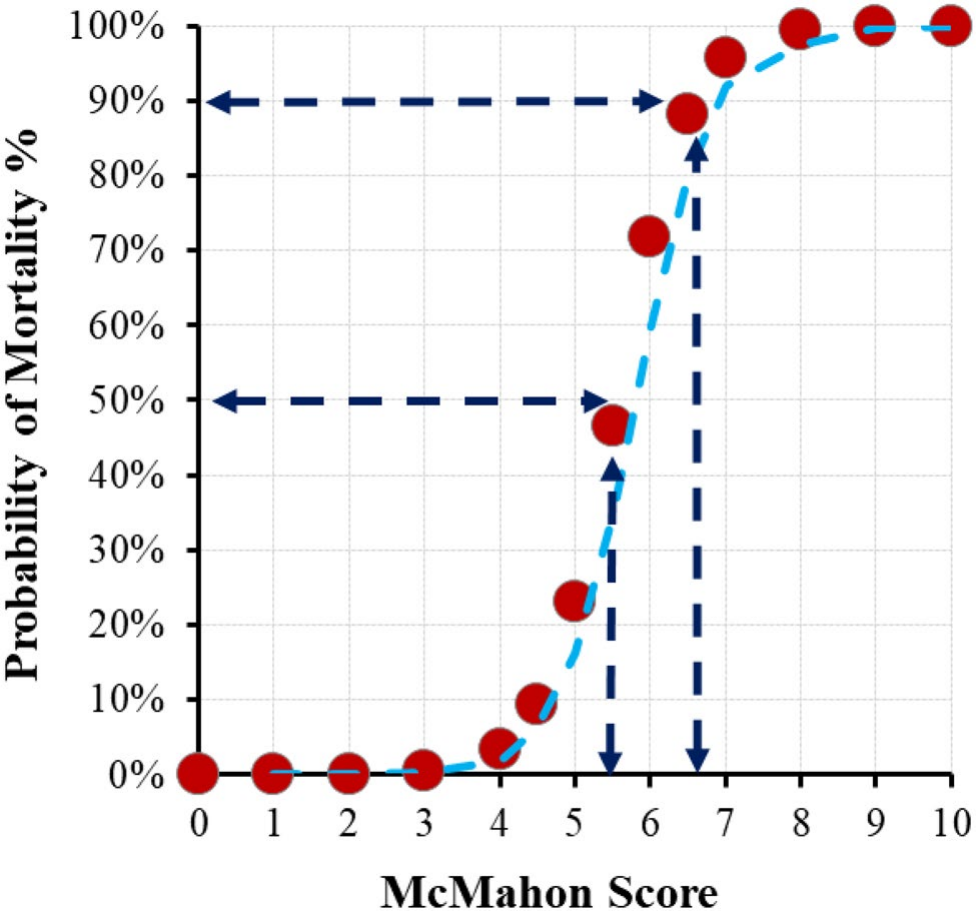
Probability of mortality (%) as a function of McMahon score with highlighted cut-off threshold and sensitivity-specificity points

No significant differences were observed in age or sex distribution between the mortality and non-mortality groups (*p* > 0.05). Non-survivors had significantly lower GCS scores (median 3.3) compared with survivors (median 15, *p* < 0.001). Similarly, the McMahon score was higher in non-survivors (median 6.5 [IQR 5.0–7.5]) than in survivors (median 3.8 [IQR 3.0–4.5]; *p* < 0.001). Laboratory findings revealed no significant differences in hemoglobin, lymphocyte, platelet, phosphate, or calcium levels between the groups (*p* > 0.05). However bicarbonate levels were significantly lower in the mortality group (*p* < 0.05). Furthermore, creatinine, creatine kinase, peak creatine kinase, and the day on which peak creatine kinase was observed were significantly higher in the mortality group compared to the non-mortality group (*p* < 0.05). The risk of AKI was also significantly higher in the mortality group (*p* < 0.05) (Table 3) (Fig. 2).

**Table 3:** Comparison of clinical and laboratory characteristics between survivor and non-survivor groups

		Survivors (*n* = 52)		Non-survivors (*n* = 14)		*P*	
		Min-Max	Median [IQR]	Min-Max	Median [IQR]		
Age		0,0-55,0	13,8-33,8	12,0-43,0	15,0-32,0	0,879	^t^
Gender	Male (42/6)					0,068	^X²^
	Female(10/8)						
GCS Score		15,0-15,0	15,0-15,0	3,0-5,0	3,0-3,0	***< 0.001***	^m^
McMahon		0,0-5,5	2,0-5,5	4,5-11,5	5,0-7,5	***< 0.001***	^m^
McMahon Score	< 6 (52/4)					***< 0.001***	^X²^
	≥ 6 (0/10)						
Hemoglobin		10,3-17,9	12,8-15,7	10,2-16,3	13,6-16,2	0,917	^t^
Albumin		2,30-5,20	3,74-4,33	2,43-4,11	2,91-4,07	***0***,***049***	^t^
Lymphocyte		0,88-6,08	1,35-3,50	1,66-7,27	1,92-5,72	0,055	^m^
Platelet		118,0-428,3	225,1-338,4	124,3-397,0	222,7-360,6	0,980	^t^
Phosphate		2,3-5,0	3,0-4,1	2,0-4,3	3,2-4,3	0,332	^m^
Calcium		7,9-10,6	9,0-9,9	1,1-10,4	8,0-9,1	0,055	^m^
Bicarbonate		18,0-28,0	20,0-24,7	4,3-18,3	9,4-17,2	***0***,***002***	^t^
Creatinine		0,33-1,10	0,63-0,86	0,98-3,22	1,04-1,54	***< 0.001***	^m^
Creatine Kinase		86-84,500	1380-3340	412-13,170	791-11900	***< 0.001***	^m^
Peak Creatine Kinase		86-84,500	1280-4370	412-50,000	1190-3191	***0***,***001***	^m^
Day Peak Creatine Kinase		1,0-3,0	1,0-1,3	1,0-7,0	1,0-4,0	***0***,***010***	^m^
% Risk of Acute Kidney Injury		1,0-8,5	2,7-5,8	5,8-50,9	7,0-17,1	***< 0.001***	^m^

^t^ Independent t test / ^m^ Mann-whitney u test / ^X²^ Chi-square test (Fischer test)

(∗):The bold and italic values indicate statistically significant results of significance

**Fig. 3:**
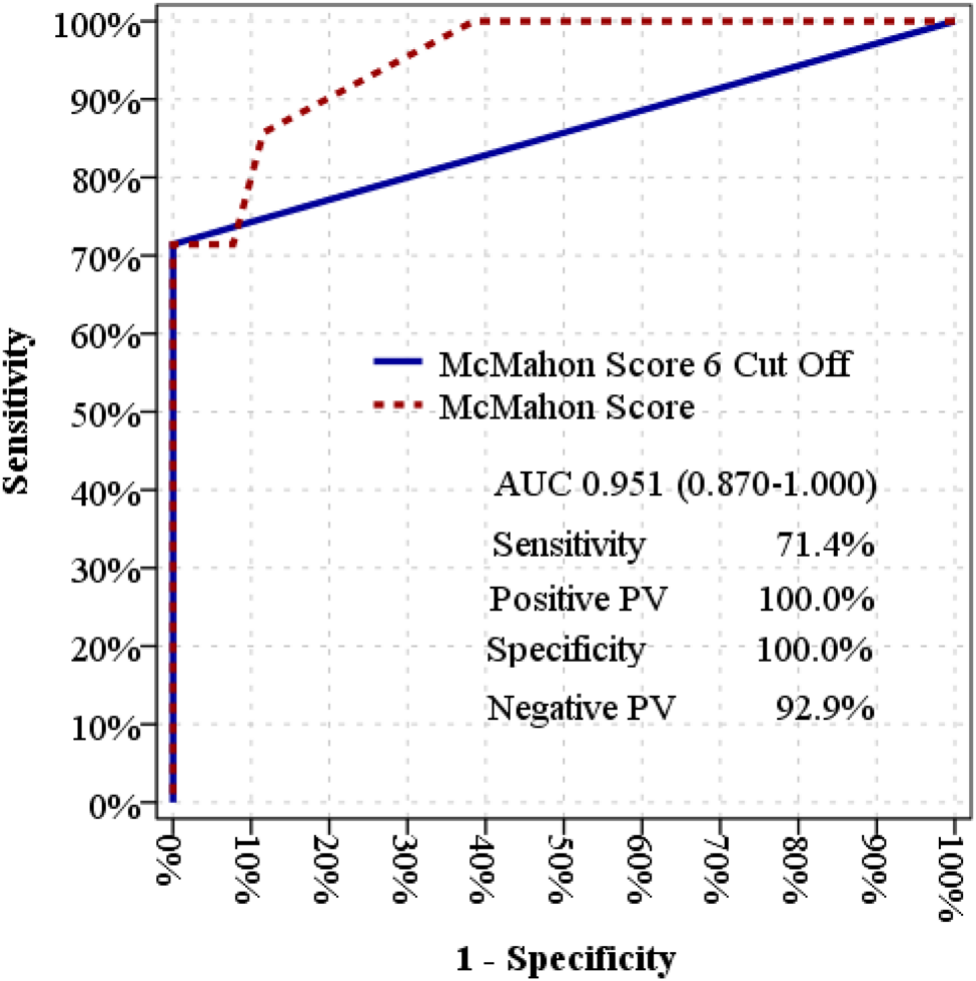
ROC curve analysis of McMahon Score and its diagnostic performance for predicting mortality

The overall effectiveness of the McMahon Score in distinguishing between mortality and non-mortality groups was found to be significant, with an area under the curve (AUC) of 0.951 (0.870–1.000). A McMahon Score cut-off value of 6 demonstrated significant efficacy in distinguishing mortality [AUC: 0.857 (0.650–1.000)]. At this cut-off value, the sensitivity, positive predictive value, specificity, and negative predictive value were determined to be 71.4%, 100.0%, 100.0%, and 92.9%, respectively (Table 4) (Fig. 3).

**Table 4:** ROC curve analysis and diagnostic performance of mcmahon score for predicting mortality

		Area Under the Curve		% 95 Confidence Interval			*p* (^∗^)
McMahon Score		0.951		0.870	-	1.000	***< 0.001***
McMahon Score 6 Cut Off		0.857		0.650	-	1.000	***0.004***
		Mortality (-)	Mortality (+)				%
McMahon Score	≤ 6	52	4	Sensitivity			71.4%
	> 6	0	10	Positive PV			100.0%
				Specificity			100.0%
				Negative PV			92.9%

(^∗^):The bold and italic values indicate statistically significant results of significance

No significant differences were observed in age or sex distribution between patients with McMahon Scores ≤ 6 and > 6 (*p* > 0.05). However, the GCS score was significantly lower in the McMahon Score > 6 group (*p* < 0.05). There were no significant differences in hemoglobin, lymphocyte, platelet, or phosphate levels between the groups (*p* > 0.05). Nevertheless, calcium, and bicarbonate levels were significantly lower in the McMahon Score > 6 group compared to the McMahon Score ≤ 6 group (*p* < 0.05). Creatinine, creatine kinase, and peak creatine kinase levels were significantly higher in the McMahon Score > 6 group (*p* < 0.05). The risk of AKI was also significantly higher in the McMahon Score > 6 group compared to the McMahon Score ≤ 6 group (*p* < 0.05). Furthermore, the mortality rate was significantly higher in the McMahon Score > 6 group than in the McMahon Score ≤ 6 group (*p* < 0.05) (Table 5).

**Table 5:** Analysis of clinical and laboratory variables based on mcmahon score categories (≤ 6 vs. > 6)

		McMahon Score ≤ 6 (*n*:56)			McMahon Score > 6 (*n*:10)			*p* (^∗^)	
		Mean ± sd/*n* (%)		Median	Mean ± sd/*n* (%)		Median		
Age		24 ± 13		21	24 ± 13		16	0.926	^t^
Gender	Male	44	78.6%		4	40.0%		0.111	^X²^
	Female	12	21.4%		6	60.0%			
GCS Score		14.1 ± 3.1		15.0	3.4 ± 0.9		3.0	***< 0.001***	^m^
Hemoglobin		14.3 ± 2.0		14.7	14.1 ± 2.4		14.3	0.851	^t^
Albumin		4.0 ± 0.6		4.1	3.4 ± 0.7		3.3	***0.036***	^t^
Lymphocyte		2.9 ± 1.8		2.3	3.5 ± 1.6		4.6	0.466	^m^
Platelet		269.9 ± 80.9		269.5	292.6 ± 81.1		259.0	0.567	^t^
Phospate		3.6 ± 0.6		3.6	3.7 ± 1.0		4.2	0.268	^m^
Calcium		9.4 ± 0.7		9.4	7.1 ± 3.4		8.3	***0.017***	^m^
Bicarbonate		21.9 ± 4.3		23.1	14.4 ± 3.8		15.6	***0.002***	^m^
Creatinine		0.8 ± 0.2		0.8	1.6 ± 0.9		1.1	***0.004***	^m^
Creatine Kinase		5780.6 ± 15590.1		1990.0	10840.0 ± 2001.4		11310.0	***0.002***	^m^
Peak Creatine Kinase		8270.4 ± 17370.1		3120.0	21290.4 ± 16210.2		14330.0	***0.004***	^m^
Day Peak Creatine Kinase		1.3 ± 0.5		1.0	3.2 ± 2.4		2.0	***0.007***	^m^
% Risk of Acute Kidney Injury		4.4 ± 2.2		4.8	21.5 ± 16.7		17.1	***< 0.001***	^m^
Mortality	(-)	52	92.9%		0	0.0%		***< 0.001***	^X²^
	(+)	4	7.1%		10	100.0%			

^t^ Independent t test / ^m^ Mann-whitney u test / ^X²^ Chi-square test (Fischer test)

(^∗^):The bold and italic values indicate statistically significant results of significance

## Discussion

In summary, our study demonstrated that patients with McMahon scores > 6 had higher mortality and AKI risk, and the score showed excellent predictive accuracy (AUC: 0.951). These findings are consistent with studies on traumatic rhabdomyolysis but extend the evidence to the rare context of lightning-induced cases. Comparing our cohort with non-lightning etiologies reported in the literature, the McMahon Score appears to demonstrate similar predictive trends: higher scores are associated with an increased risk of AKI and adverse outcomes. Nevertheless, lightning injuries possess unique pathophysiologic features (e.g., combined electrical and thermal tissue effects, concomitant neurologic/cardiac injury) that may modify risk profiles. Accordingly, while our findings support the potential applicability of the McMahon Score in lightning-induced rhabdomyolysis, external validation in larger, multicenter cohorts is warranted before broader generalization. Lightning strike-induced rhabdomyolysis is a rare condition that can result in severe complications [3]. The median age of the patients in the study was 21 years, indicating that lightning strikes predominantly affect younger populations. The literature suggests that environmental traumas like lightning strikes frequently affect individuals who work or are active outdoors, with younger age and male gender being more common in this group [11, 12]. In our study, 72.7% of participants were male, which aligns with findings from other studies on lightning strikes and rhabdomyolysis [4]. The lack of a significant effect of age and gender on mortality (*p* > 0.05) suggests that these demographic factors play a limited role as prognostic markers in rhabdomyolysis cases caused by lightning strikes. In the literature, conflicting data exist regarding the impact of age and gender on trauma-related mortality. For instance, some studies have reported that older age is associated with poorer prognosis, while younger age groups demonstrate better physical resilience and response to treatment [13, 14]. The predominance of a younger population in our study may have contributed to the lack of significant age-related effects.

Although GCS was significantly lower in non-survivors, the primary prognostic tool evaluated in our study was the McMahon score, which demonstrated stronger predictive value for mortality and AKI [15, 16]. The median McMahon score was 3.8 [IQR: 3.0–4.5] in survivors compared with 6.5 [IQR: 5.0–7.5] in non-survivors, and this difference was statistically significant (*p* < 0.001, Mann–Whitney U test). Particularly in complex incidents like lightning strikes, lower GCS scores are associated with an increased risk of neurological damage and systemic organ dysfunction [17]. The McMahon Score demonstrated strong predictive ability in our cohort; however, these findings should be interpreted with caution and require validation in larger, multicenter studies. The area under the curve (AUC) for the score was calculated as 0.951, and a cutoff value of 6 demonstrated high sensitivity (71.4%) and specificity (100%) in mortality prediction. Although similar predictive trends have been observed in rhabdomyolysis of different etiologies, our study affirms the utility of the McMahon Score in a rare and underrepresented clinical context. In the literature, the McMahon Score is primarily used to predict acute kidney injury (AKI) secondary to rhabdomyolysis [4]. Significant correlations were identified between the McMahon Score and biochemical parameters such as bicarbonate, creatinine, and creatine kinase. In the group with a McMahon Score > 6, low albumin levels were associated with poor prognosis and increased mortality risk. Albumin, although not a component of the McMahon score, was significantly lower in non-survivors and may reflect poorer overall clinical status [8, 18]. Low bicarbonate levels, indicative of metabolic acidosis, were also associated with poor prognosis in the group with a McMahon Score > 6. The literature similarly notes that metabolic acidosis exacerbates organ dysfunction in rhabdomyolysis cases [19]. Elevated creatine kinase levels reflect the severity of rhabdomyolysis and the extent of muscle damage. In the group with a McMahon Score > 6, significantly higher creatine kinase levels indicated a more critical clinical condition [8]. AKI secondary to rhabdomyolysis occurs due to myoglobin accumulation in renal tubules and the induction of oxidative stress. The risk of AKI was significantly higher in the group with a McMahon Score > 6 (*p* < 0.05). Early aggressive fluid therapy has been reported in the literature as crucial in preventing AKI secondary to rhabdomyolysis [20]. The differences between our findings and previous studies may be explained by the unique pathophysiological mechanisms of lightning injuries, the younger age of our cohort, and the relatively small sample size compared with trauma-related rhabdomyolysis studies. Previous research has primarily focused on traumatic rhabdomyolysis [4, 9, 21]. While earlier studies have validated the effectiveness of the McMahon Score in predicting AKI, limited data exist on its role in mortality prediction [4, 7, 8]. This study fills this gap and makes a significant contribution to the literature. Similarly, Comoğlu et al. (2025) proposed a novel scoring system for predicting the need for renal replacement therapy in crush injury patients. Although not directly related to lightning-induced rhabdomyolysis, this study underscores the methodological importance of developing simple and effective prognostic tools in trauma-related conditions [22]. Based on the findings of this study, our emergency department has emphasized early identification of patients with McMahon scores > 6, with immediate initiation of aggressive fluid therapy and close monitoring for AKI. These measures have been integrated into local protocols to improve outcomes in lightning-induced rhabdomyolysis, and sharing these experiences may help other centers facing similar rare but severe cases. While our findings support the potential clinical utility of the McMahon Score in lightning-induced rhabdomyolysis, they should be interpreted with caution, given the rarity of the condition and the need for external validation.

## Limitations

This study has certain limitations due to its retrospective design, which may be subject to missing or incomplete records. Additionally, the single-center nature of the study and the limited sample size restrict the generalizability of the findings to a broader population. Future studies with larger sample groups and validation across multiple centers are essential to confirm these results. Although our study does not propose new treatment strategies or mechanisms, it provides one of the largest data sets focused specifically on lightning-induced rhabdomyolysis and supports the external validation of an established prognostic tool in this underexplored context.

## Conclusions

This study adds to the limited literature evaluating the utility of the McMahon Score in lightning-induced rhabdomyolysis. Our findings suggest that the McMahon Score has prognostic value for mortality and rhabdomyolysis-associated complications. Its simplicity and rapid applicability may provide practical support for early assessment and decision-making in the emergency department. However, these results should be interpreted with caution due to the retrospective single-center design and small sample size, and larger multicenter studies are warranted to confirm these findings.

## Data Availability

All data relevant to the study are included in the article.

## References

[1] Hebert JF, Burfeind KG, Malinoski D, Hutchens MP. Molecular mechanisms of Rhabdomyolysis-Induced kidney injury: from bench to bedside. Kidney Int Rep. 2022;8(1):17–29. 10.1016/j.ekir.2022.09.026. 36644345 10.1016/j.ekir.2022.09.026 PMC9831947

[2] El Ghoch M, Calugi S, Dalle Grave R. Management of severe rhabdomyolysis and Exercise-Associated hyponatremia in a female with anorexia nervosa and excessive compulsive exercising. Case Rep Med. 2016;2016:8194160. 10.1155/2016/8194160. 27721832 10.1155/2016/8194160 PMC5046051

[3] Navarrete N. Severe rhabdomyolysis without renal injury associated with lightning strike. J Burn Care Res. 2013;34(3):e209–12. 10.1097/BCR.0b013e31825adc98. 22929530 10.1097/BCR.0b013e31825adc98

[4] McMahon GM, Zeng X, Waikar SS. A risk prediction score for kidney failure or mortality in rhabdomyolysis. JAMA Intern Med. 2013;173(19):1821–8. 10.1001/jamainternmed.2013.9774. 24000014 10.1001/jamainternmed.2013.9774 PMC5152583

[5] Parammal Alikutty J, Raj A, Soofi SK, et al. Rhabdomyolysis-Induced acute kidney injury (AKI) in a young bodybuilder: A case report. Cureus. 2023;15(2):e34625. 10.7759/cureus.34625. Published 2023 Feb 4. 36891010 10.7759/cureus.34625 PMC9987342

[6] Okafor UV. Lightning injuries and acute renal failure: a review. Ren Fail. 2005;27(2):129–34. 10.1081/JDI-200048216. 15807175

[7] Chavez LO, Leon M, Einav S, Varon J. Beyond muscle destruction: a systematic review of rhabdomyolysis for clinical practice. Crit Care. 2016;20(1):135. 10.1186/s13054-016-1314-5. 27301374 10.1186/s13054-016-1314-5 PMC4908773

[8] Simpson JP, Taylor A, Sudhan N, Menon DK, Lavinio A. Rhabdomyolysis and acute kidney injury: creatine kinase as a prognostic marker and validation of the mcmahon score in a 10-year cohort. Eur J Anaesthesiol. 2016;33(12):906–12. 10.1097/EJA.0000000000000490. 27259093 10.1097/EJA.0000000000000490

[9] Yaman M, Şen A, Durgun HM, et al. Evaluating the mcmahon score for predicting mortality in earthquake-induced rhabdomyolysis: a retrospective study. Postgrad Med J. 2024;101(1191):45–9. 10.1093/postmj/qgae103. 39140606 10.1093/postmj/qgae103

[10] Subashri M, Sujit S, Thirumalvalavan K, Poongodi A, Srinivasaprasad ND, Edwin Fernando M. Rhabdomyolysis-associated acute kidney injury. Indian J Nephrol. 2023;33(2):114–8. 10.4103/ijn.ijn_247_21. 37234438 10.4103/ijn.ijn_247_21 PMC10208535

[11] Gowing R, Jain MK. Injury patterns and outcomes associated with elderly trauma victims in Kingston, Ontario. Can J Surg. 2007;50(6):437–44. 10.1016/j.elstat.2006.09.016. 18053371 PMC2386230

[12] Lat TI, McGraw MK, White HD. Gender differences in critical illness and critical care research. Clin Chest Med. 2021;42(3):543–55. 10.1016/j.ccm.2021.04.012. 34353458 10.1016/j.ccm.2021.04.012 PMC8328243

[13] Mathew M, Pillai SCB. Clinical outcomes of rhabdomyolysis & validation of mcmahon score for risk prediction. Indian J Med Res. 2024;159(1):102–8. 10.4103/ijmr.ijmr_2733_21. 38391136 10.4103/ijmr.ijmr_2733_21 PMC10954106

[14] Vincent JL, Dubois MJ, Navickis RJ, Wilkes MM. Hypoalbuminemia in acute illness: is there a rationale for intervention? A meta-analysis of cohort studies and controlled trials. Ann Surg. 2003;237(3):319–34. 10.1097/01.SLA.0000055547.93484.87. 12616115 10.1097/01.SLA.0000055547.93484.87 PMC1514323

[15] Grote S, Böcker W, Mutschler W, Bouillon B, Lefering R. Diagnostic value of the Glasgow coma scale for traumatic brain injury in 18,002 patients with severe multiple injuries. J Neurotrauma. 2011;28(4):527–34. 10.1089/neu.2010.1433. 21265592 10.1089/neu.2010.1433

[16] Van Ruler R, Eikendal T, Kooij FO, Tan ECTH. A shocking injury: A clinical review of lightning injuries highlighting pitfalls and a treatment protocol. Injury. 2022;53(10):3070–7. 10.1016/j.injury.2022.08.024. 36038387 10.1016/j.injury.2022.08.024

[17] Andrews CJ, Reisner AD. Neurological and neuropsychological consequences of electrical and lightning shock: review and theories of causation. Neural Regen Res. 2017;12(5):677–86. 10.4103/1673-5374.206636. 28616016 10.4103/1673-5374.206636 PMC5461597

[18] Schrezenmeier EV, Barasch J, Budde K, Westhoff T, Schmidt-Ott KM. Biomarkers in acute kidney injury - pathophysiological basis and clinical performance. Acta Physiol (Oxf). 2017;219(3):554–72. 10.1111/apha.12764. 27474473 10.1111/apha.12764 PMC5575831

[19] Wen K, Huang Y, Guo Q, et al. Predicting risk factors of acute kidney injury in the first 7 days after admission: analysis of a group of critically ill patients. Cardiovasc Ther. 2022;2022:1407563. 10.1155/2022/1407563. Published 2022 Dec 21. 36628120 10.1155/2022/1407563 PMC9797299

[20] Kim HW, Kim S, Ohn JH, et al. Role of bicarbonate and volume therapy in the prevention of acute kidney injury in rhabdomyolysis: a retrospective propensity score-matched cohort study. Kidney Res Clin Pract. 2022;41(3):310–21. 10.23876/j.krcp.21.093. 34974654 10.23876/j.krcp.21.093 PMC9184844

[21] Ware LB. Biomarkers in critical illness: new insights and challenges for the future. Am J Respir Crit Care Med. 2017;196(8):944–5. 10.1164/rccm.201704-0831ED. 28475361 10.1164/rccm.201704-0831ED

[22] Comoğlu M, Acehan F, İnan O, et al. A new score predicting renal replacement therapy in patients with crush injuries: analysis of a major earthquake. Am J Emerg Med. 2025;87:1–7. 10.1016/j.ajem.2024.10.031. 39447493 10.1016/j.ajem.2024.10.031

